# Diversity, seasonal abundance, and environmental drivers of chaetognath populations in North Inlet Estuary, South Carolina, USA

**DOI:** 10.1002/ece3.10151

**Published:** 2023-05-30

**Authors:** Sarah E. Stone, Joshua P. Stone

**Affiliations:** ^1^ College of Earth, Ocean, and Atmospheric Sciences Oregon State University Corvallis Oregon USA; ^2^ Department of Biological Sciences University of South Carolina Columbia South Carolina USA

**Keywords:** chaetognath, estuary, marine food webs, seasonality, zooplankton

## Abstract

Chaetognaths (Phylum: Chaetognatha) are one of the most abundant phyla of zooplankton worldwide and play an important role in marine trophic interactions. Although the role of chaetognaths in global ecosystems is well understood, the spatial variation and environmental drivers of estuarine chaetognath populations is poorly understood. To provide the first known record of chaetognath species composition in a coastal estuary in the south‐eastern USA, chaetognaths were identified and quantified from zooplankton samples collected on a monthly basis in 2019 and 2020 from North Inlet Estuary in South Carolina. *Parasagitta tenuis* was the most abundant species of the five found, making up 33% of total abundance. The egg presence of these chaetognaths was further analyzed to gauge reproductive cycles. Abundance and egg presence were compared with surface and bottom measurements of temperature, salinity, and dissolved oxygen levels to determine the driving abiotic factors behind chaetognath's seasonal variability and reproductive cycles. Temperature, salinity, and dissolved oxygen all had low (*r* < ±.29), non‐significant correlations with abundance. Chaetognath egg production was most significantly associated with dissolved oxygen (*p* < .001) and seasonal changes in temperature (*p* < .001). Our initial findings indicate the continued abundance of chaetognath in a local estuary are dependent on abiotic factors that are strongly influenced by a changing climate.

## Graphical abstract

In this manuscript we quantified and identified chaetognaths from zooplankton samples collected on a monthly basis in a coastal estuary. We assess the effects of temperature, salinity, and dissolved oxygen on chaetognath's seasonality, species composition, and reproductive timing. Our findings highlight the potential influence of environmental factors on chaetognath abundance and reproductive timing, addressing a knowledge gap in the ecology of this important zooplankton phyla's seasonality.

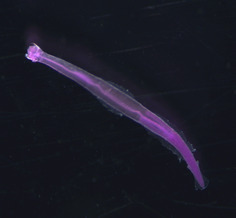

## 1 INTRODUCTION

**FIGURE 1:**
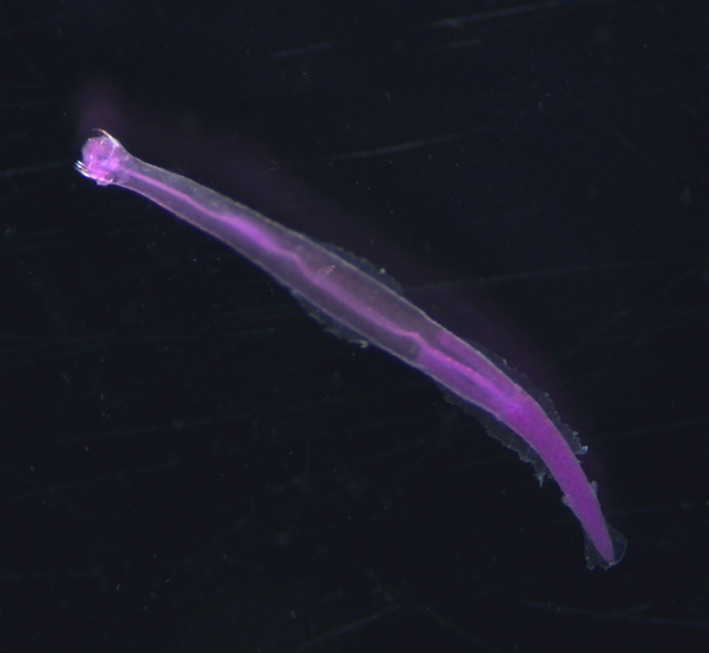
Preserved and stained *Parasagitta tenuis* collected from North Inlet Estuary.

Chaetognaths (Phylum: Chaetognatha) are one of the most abundant phyla of mesozooplankton, second in number only to copepods (Feigenbaum & Maris, 1984; Terazaki, 1998; Froneman et al., 1998). The 132 species comprising the Chaetognaths phylum vary in size from 2 to 120 mm (Ahyong et al., 2023), with bodies that are generally transparent and bilaterally symmetrical (Figure 1). Chaetognaths primarily prey on copepods and can have abundances as high as 30% of copepod wet weight biomass globally (Terazaki, 1998). Due to their high abundance, chaetognaths play a significant role in transferring energy to higher trophic levels within marine food webs, as they prey on other mesozooplankton, macroplankton, and small vertebrates such as larval fish (Lebour, 1923; Alvariño, 1975; Kuhlmann, 1977; Baier & Purcell, 1997; Coston‐Clements et al., 2009). Several studies have suggested chaetognaths can cause significant mortality in fish larvae, due to their high abundances and the documented presence of larval fish in their guts (reviewed in Alvariño, 1985; Baier & Purcell, 1997).

Chaetognaths can be found within coastal, neritic, and oceanic habitats throughout the world. Most species are pelagic and exhibit diel vertical migration related to prey availability and resource partitioning (Gibbons, 1992; Giesecke & González, 2004; Johnson et al., 2006; Kehayias & Kourouvakalis, 2010; Stuart & Verheye, 1991; Terazaki, 1996). Chaetognaths have weak osmoregulatory functioning, which forces them to occupy narrow salinity ranges within select oceanic zones and water masses (Bieri, 1959; Coston‐Clements et al., 2009; Owre, 1960; Pierce, 1951; Pierce & Wass, 1962; Ulloa et al., 2000). Each species has different environmental tolerances and preferences, and understanding these conditions provides valuable information regarding the associated water masses (Owre, 1960; Pierce & Wass, 1962).

Chaetognaths reproduce sexually and are hermaphroditic, with individuals possessing both a pair of seminal vesicles and receptacles (Ghirardelli, 1969; Goto & Yoshida, 1985; Pierrot‐Bults, 1976). Upon maturation of fertilized eggs, the eggs are expelled from the individual, where they attach to vegetation or develop within marsupial sacs (Terazaki & Miller, 1982). Chaetognaths surpass the larval stage and emerge from their eggs as juveniles. This development occurs quickly, with some species developing from fertilization to juvenile within several days (Goto & Yoshida, 1997; Margulis and Chapman, 2010). Alvariño (1990) suggested chaetognath egg development and seasonal laying times may be dependent on temperature, with laying occurring earlier in the day on warm days and most species breeding primarily in spring, with breeding occurring year‐round in tropical waters.

While research into the ecology of chaetognaths, specifically related to their seasonality (Gilmartin et al., 2020; Lozano‐Cobo et al., 2017; Nogueira Júnior et al., 2019; Tse et al., 2008), has increased over the last few decades, there is still a gap in our scientific understanding of estuarine species' geographic distribution and the environmental factors which drive their abundance and reproduction. O'Brien (1977) and Tiselius and Peterson (1986) suggested chaetognath distribution and species composition depend on temperature and salinity levels. Both Lonsdale and Coull (1977) and Houser and Allen (1996) verified the presence of chaetognaths in North Inlet Estuary, but did not identify them to species level. Johnson and Allen (2012) provided details on the behavior, morphology, and range of zooplankton species within the coastal zone of the southeastern USA, which included six species of chaetognath (*Parasagitta elegans* (Verrill, 1873; formerly known as *Sagitta elegans*), *Parasagitta tenuis* (Conant, 1896; formerly known as *Sagitta tenuis*), *Flaccisagitta enflata* (Grassi, 1881; formerly known as *Sagitta enflata*), *Ferosagitta hispida* (Conant, 1895; formerly known as *Sagitta hispida*), *Sagitta helenae*, and *Sagitta bipunctata*). *Parasagitta tenuis*, *Parasagitta elegans*, *Sagitta helenae*, and *Ferosagitta hispida* can primarily be found in neritic waters, whereas *S. bipunctata* is a predominantly oceanic species (Avila & Cadena, 1999; Owre, 1960; Thuesen et al., 1993; Tovar et al., 2009). Previous research on chaetognaths in the southeastern USA has focused on determining species composition and the reproductive seasonality of chaetognaths (Owre, 1960; Pierce & Wass, 1962), as well as the interaction between chaetognaths and larval fish (Coston‐Clements et al., 2009).

As chaetognath abundance in North Inlet Estuary has been previously shown to have a positive relationship with temperature (Houser & Allen, 1996), we hypothesize temperature to act as a tracer for abundance and that chaetognath abundance and egg presence will demonstrate similar seasonality following the climate of the estuary. There are several other factors likely impacting chaetognath seasonality, including prey availability and predator abundance. We hypothesize the presence and fluctuating abundances of chaetognath in North Inlet Estuary has the potential to alter copepod stock. While this study does not aim to assess actualized impacts of chaetognaths on copepod stocks in North Inlet Estuary, through the use of ranges of chaetognath digestion rates and average numbers of prey per chaetognath found in previous literature, as well as previous data on copepod stock within the estuary, and chaetognath abundances found in this study, we can make estimates regarding copepod predation in the system.

The purpose of this study is to provide the first known record of chaetognath species composition within the North Inlet‐Winyah Bay estuary, while contributing a local dataset regarding their reproductive cycle and its driving forces. Understanding the seasonality of chaetognath abundance and egg presence in the North Inlet Estuary can aid in our understanding of marine food webs, as well as chaetognath's seasonal effects on the copepod population in the system.

## 2 METHODS

### 2.1 Description of study area

The study was conducted in North Inlet Estuary, in Georgetown County, South Carolina (Figure 1), a barrier‐island‐bounded system with temperate, high salinity water that is primarily surrounded by a *Spartina alterniflora* marsh (Allen et al., 2008). Approximately 90% of the estuary's watershed is protected by a private foundation and has remained undeveloped over the past 200 years, keeping the estuary relatively free from local anthropogenic impacts (Allen et al., 2014).

The sampling site is roughly 2 km from the ocean and is at the confluence of Town, Bread and Butter, and Clambank creeks (79.1877° W, 33.3320° N). The depth in the western portion of the channel where sampling occurred is ~2 m at low tide, with an average semidiurnal tidal range of 1–2 m. The bottom is a sand‐mud matrix and due to the shallow water depth and high tidal currents, the creek is generally well mixed and vertically homogenous with respect to dissolved substances. Additionally, due to the high input of oceanic waters and protected watershed, in the past nutrient concentrations in the estuary were low and water quality was high (Allen et al., 2008; Dame et al., 1986).

The salinity of North Inlet Estuary is primarily controlled by the semidiurnal exchange of water with the coastal ocean, with small amounts of freshwater input from a forested watershed. Salinity in North Inlet usually remains high (30–35) except when there are influxes from heavy local rainfall, which can reduce salinity concentrations to below 20 for several days. Similarly, when the brackish water from Winyah Bay to the south moves into the North Inlet system, salinity levels can be reduced significantly (15–25) for a time span of weeks to months (Allen et al., 2008; Traynum & Styles, 2007).

**FIGURE 2:**
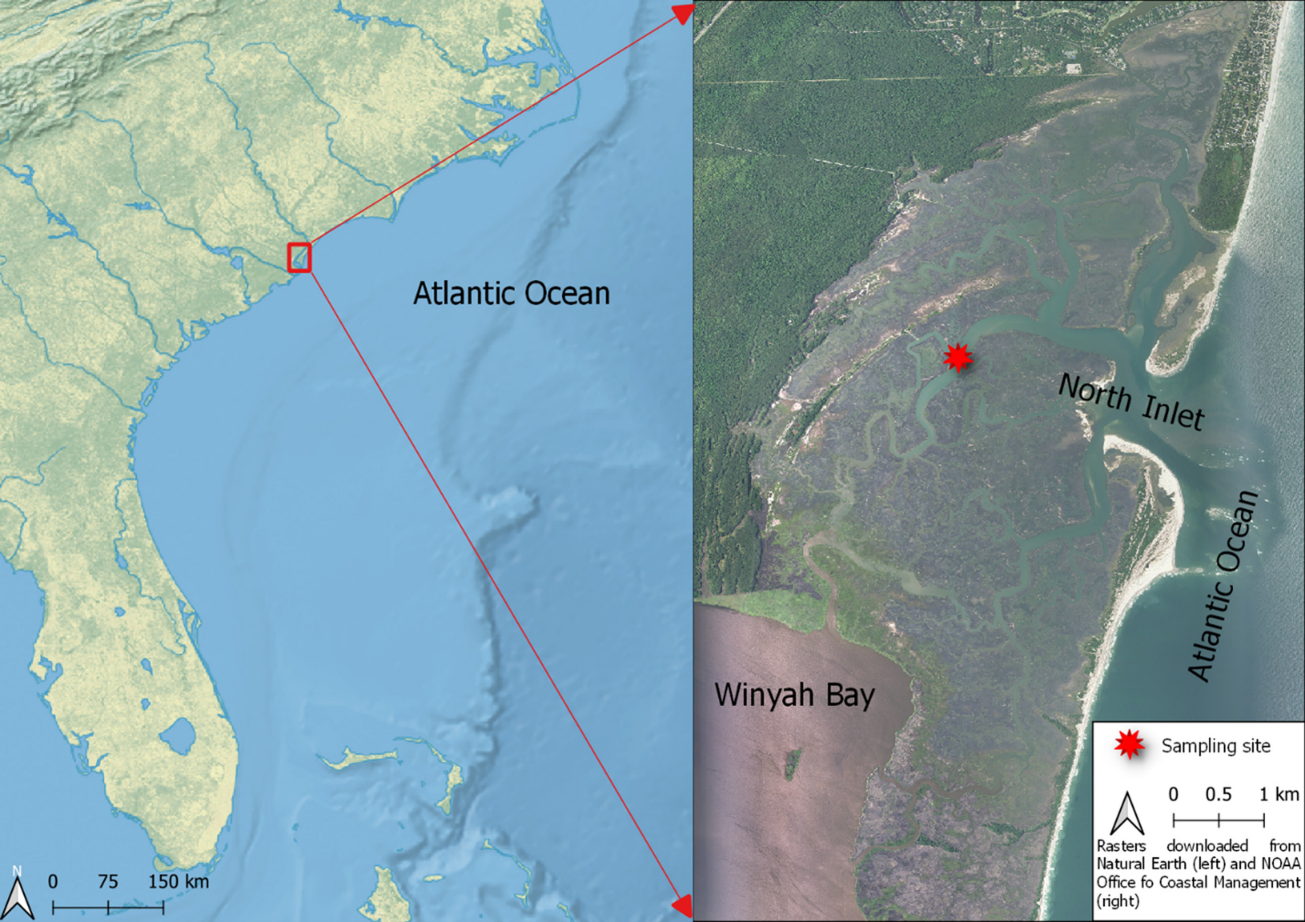
Map of the study site location. Left: Southeastern United States, Right: North Inlet Estuary and sampling location.

Estuarine systems are highly dynamic with respect to their hydrology, nutrient cycling, and biotic resource management. Estuaries' complex mixture of tidal saltwater and freshwater runoff are shaped by certain climatic forcing features, such as temperature, amount of rainfall, and wind patterns (Paerl et al., 2010). The North Inlet is a system vulnerable to the extreme effects of these long‐term changes to the natural system and has shown an increase in average temperatures of 1.5°C over the past 40 years (Allen et al., 2014) (Figure 2).

### 2.2 Sampling design

The sampling design followed the methods of Houser and Allen (1996). Zooplankton samples were collected during one tow between 1000 and 14,000 h each month during outgoing mid‐tide from November 2019 to November 2020. Tows were carried out horizontally, 20 m from the marsh bank, parallel to the shore, and in the direction of the ebbing tide. Each tow lasted roughly 5 min and covered 200 m. The tows were conducted aboard a boat with an epibenthic sled equipped with a 365‐μm mesh net, a mouth opening of 50 cm wide by 35 cm high, and with a general oceanics flowmeter attached to the net's mouth. During the tow, the net mouth was just above the bottom, ~1.5 m below the surface water. Surface (~0.5 m below surface water) and bottom (~1.5 m below surface water) salinity, temperature, and dissolved oxygen were measured from an anchored boat using a YSI Pro 2030 Sonde.

Upon collection, samples were immediately preserved in 4% borax‐buffered formaldehyde‐seawater solution. Samples were then processed in the lab using a dissecting microscope at 10–40× magnification. For samples with high volumes of animals and small detritus, a Folsom splitter was used to subsample. All chaetognaths found were identified to species using Johnson and Allen (2012), and subsequently counted and classified by egg presence. Egg presence was quantified by the visual presence of eggs. If egg presence was visible, then the chaetognath was counted as having eggs present, whereas if no eggs were visible, the chaetognath was classified as having no eggs present.

### 2.3 Data analysis and statistics

Abundance values for the number of individuals with eggs present, no eggs present, and total chaetognaths were converted into the volume of animals per m^3^ using the 0.175 m^2^ mouth opening and flowmeter readings for each sample. Unidentifiable fragments of chaetognaths found were labeled as “Unknown Chaetognath” (UK) and included in statistical analyses of total chaetognath abundance. Two samples were analyzed in November and March, respectively; the chaetognath abundance and accompanying environmental data were averaged for the two samples within each month.

All statistical analyses were done using R Statistical Software (v4.2.3; R Core Team, 2023). Pearson correlation coefficients were used to determine the relationship between chaetognath abundance, egg presence, and environmental variables (salinity, temperature, and dissolved oxygen) at both surface and bottom depths. To visualize environmental influences on chaetognath community composition, we conducted a redundancy analysis (RDA).

To estimate chaetognath's impact on copepod stock in North Inlet Estuary, we used the formula derived by Bajkov (1935) as seen in Feigenbaum & Maris (1984) and Baier & Purcell (1997):

$${\mathrm{FR}}_{N}=\frac{\mathrm{NPC}\times T}{\mathrm{DT}}$$

where FR_N_ is the daily feeding rate (prey per chaetognath per day), NPC is the prey per chaetognath, *T* is the time period in hours, and DT is digestion time in hours. *T* = 24 was used for all calculations. As consumption and digestion times can vary between species (Feigenbaum & Maris, 1984; Baier & Purcell, 1997), we utilized average values for digestion times and prey per chaetognath values for only *S. helenae* and *F. hispida* as these were the only species found in both this study and Baier and Purcell (1997). Digestion time was calculated by observing isolated chaetognaths with prey and recording the times (in 15 min intervals) of initial isolation, prey time, and position within the gut (Baier & Purcell, 1997). Chaetognaths were observed until the prey was no longer visible in the gut, then digestion time estimates were multiplied by two to account for digestion that may have been occurring prior to observation (Baier & Purcell, 1997). The average a mean and standard error of the mean digestion time for *S. helenae* and *F. hispida* was 269.2 ± 74.5 min (*n* = 82) (Baier & Purcell, 1997). Prey per chaetognath was calculated by observing the number of prey organisms in individual chaetognath's gut, multiplying this value by two to account for prey loss from gut evacuation, and calculating the mean across analyzed samples (Baier & Purcell, 1997). The average prey per chaetognath for *S. helenae* and *F. hispida* (*n* = 82) was 2.151 (Baier and Purcell, 1997).

## 3 RESULTS

### 3.1 Environmental conditions

As typical of estuarine systems, the environmental conditions of the sampling site varied seasonally. Water temperatures measured during the sampling sessions ranged from 8.9°C to 30.6°C over the course of the sampling year. Salinity values ranged from 19.8 to 34.2, with an average bottom salinity of 29.3 and an average surface salinity of 27.4. Dissolved oxygen concentrations ranged from 2.86 to 11.26 mg/L.

### 3.2 Abundance and egg presence

**FIGURE 3:**
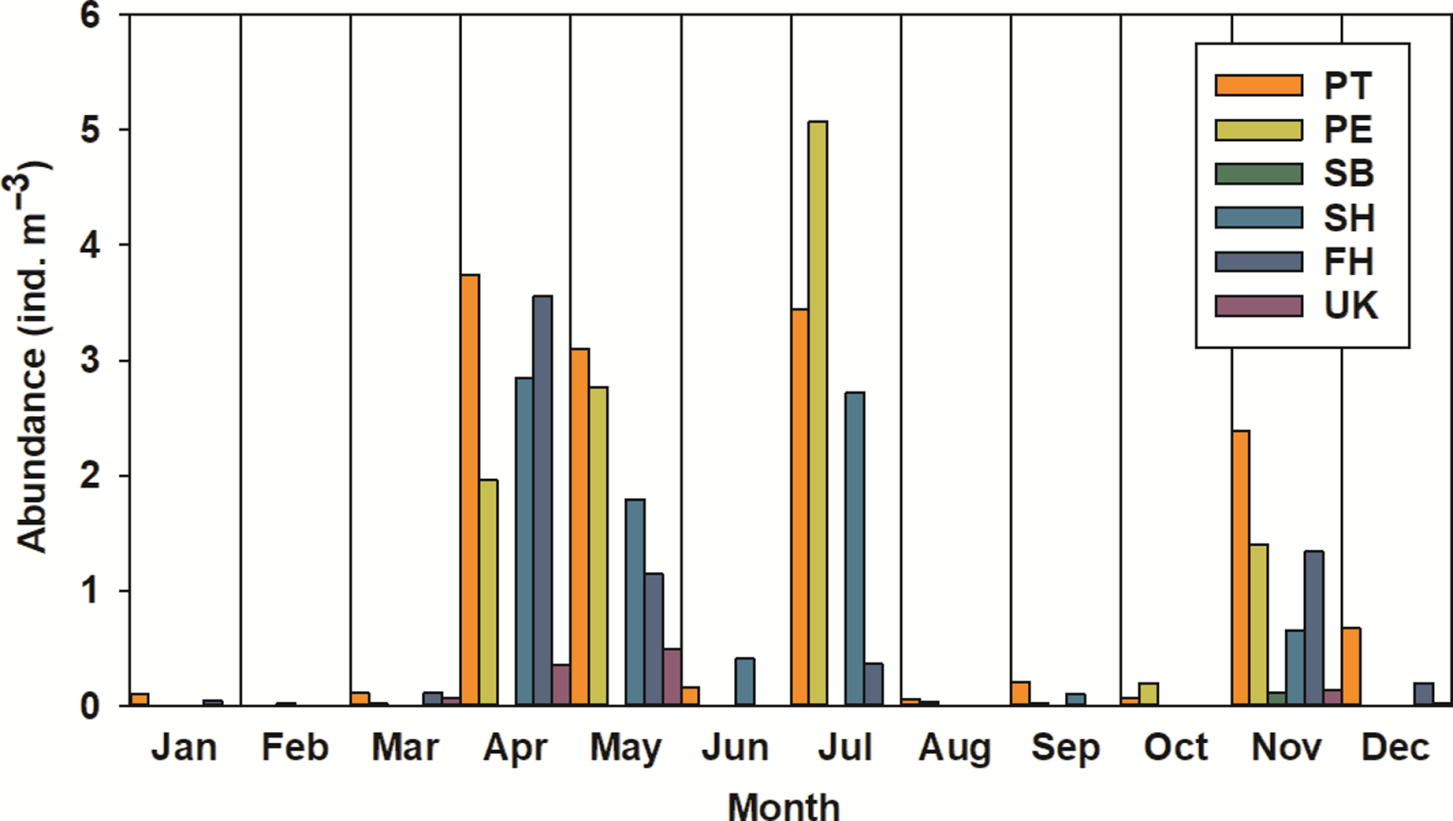
Abundance of total chaetognaths (in individuals per m^−3^) in each month of the year (*n* = 708). FH, *Ferosagitta hispida*; PE, *Parasagitta elegans*; PT, *Parasagitta tenuis*; SB, *Sagitta bipunctata*; SH, *Sagitta helenae*; UK, Unknown chaetognath.

Chaetognath abundance averaged across all samples was 3.5 individuals per m^3^. Five species of chaetognaths were found to reside in North Inlet Estuary: *Parasagitta tenuis*, *Parasagitta elegans*, *Sagitta helenae*, *Ferosagitta hispida*, and *S. bipunctata*. *P. tenuis* was the most abundant species collected, contributing to 33% of total chaetognath abundance. We observed high monthly fluctuations in chaetognath abundance (Figure 3), with the highest values measured in April, May, July, and November. Abundance was low in first 3 months of the year, as well as in late summer/early fall. The species *S. bipunctata* was found only in the months of February and November (Figure 3). *P. tenuis* and *F. hispida* both had the highest abundances in April, whereas *P. elegans* abundances were highest in July. *S. helenae* also had the highest abundances in April and July.

**FIGURE 4:**
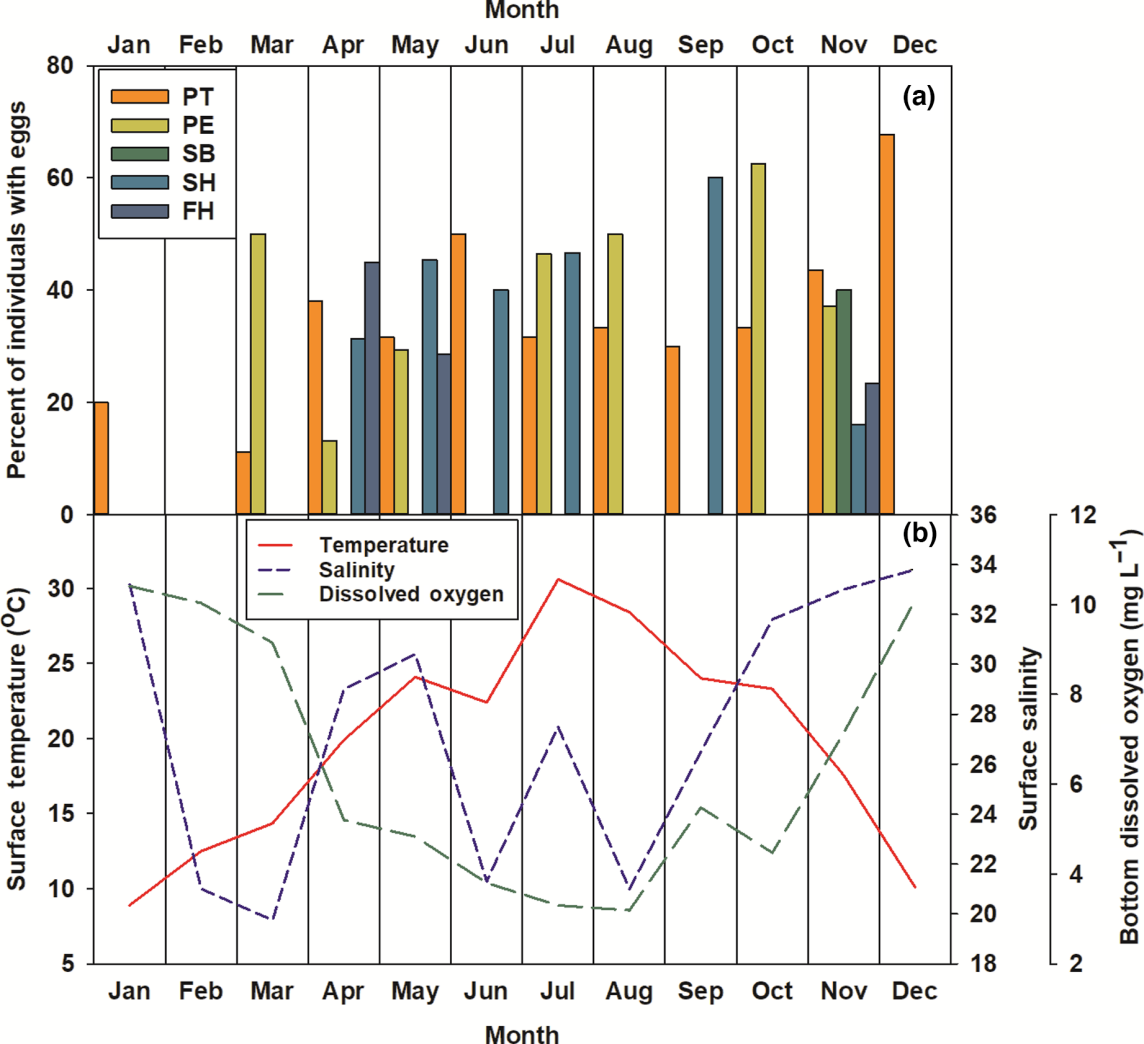
(a) The percentage of individuals with eggs present per month and species (*n* = 708). FH, *Ferosagitta hispida*; PE, *Parasagitta elegans*; PT, *Parasagitta tenuis*; SB, *Sagitta bipunctata*; SH, *Sagitta helenae*; UK, Unknown chaetognath. (b) Surface temperature (*r* = .455; *t*(70) = 4.2748; *p* < .001; 95% CI [0.2496, 0.6212])), surface salinity (*r* = .274; *t*(70) = 2.3868; *p* = .0197; 95% CI [0.0456, 04758]), and bottom dissolved oxygen (*r* = −.536; *p* < .001; *t*(70) = −5.317; 95% CI [−0.6831, −0.3479]) measurements per month.

On average, 33% of chaetognaths analyzed had developed reproductive systems enough for eggs to be visible. Egg presence of three taxa (*P. elegans*, *P. tenuis*, *S. helenae*) gradually increased throughout the year, peaking in autumn (*P. elegans*, *S. helenae*) and winter (*P. tenuis*) (Figure 4). The highest percentage of total individuals with eggs, belonging to known taxa, occurred in October (54.5%). The highest percentage of total individuals with eggs present occurred in May for unknown taxa (“UK”). *P. tenuis* had the highest egg percentage in December (67.7%), *P. elegans* in October (62.5%), *S. helenae* in September (60%), and *F. hispida* in April (45%) (Figure 4).

**FIGURE 5:**
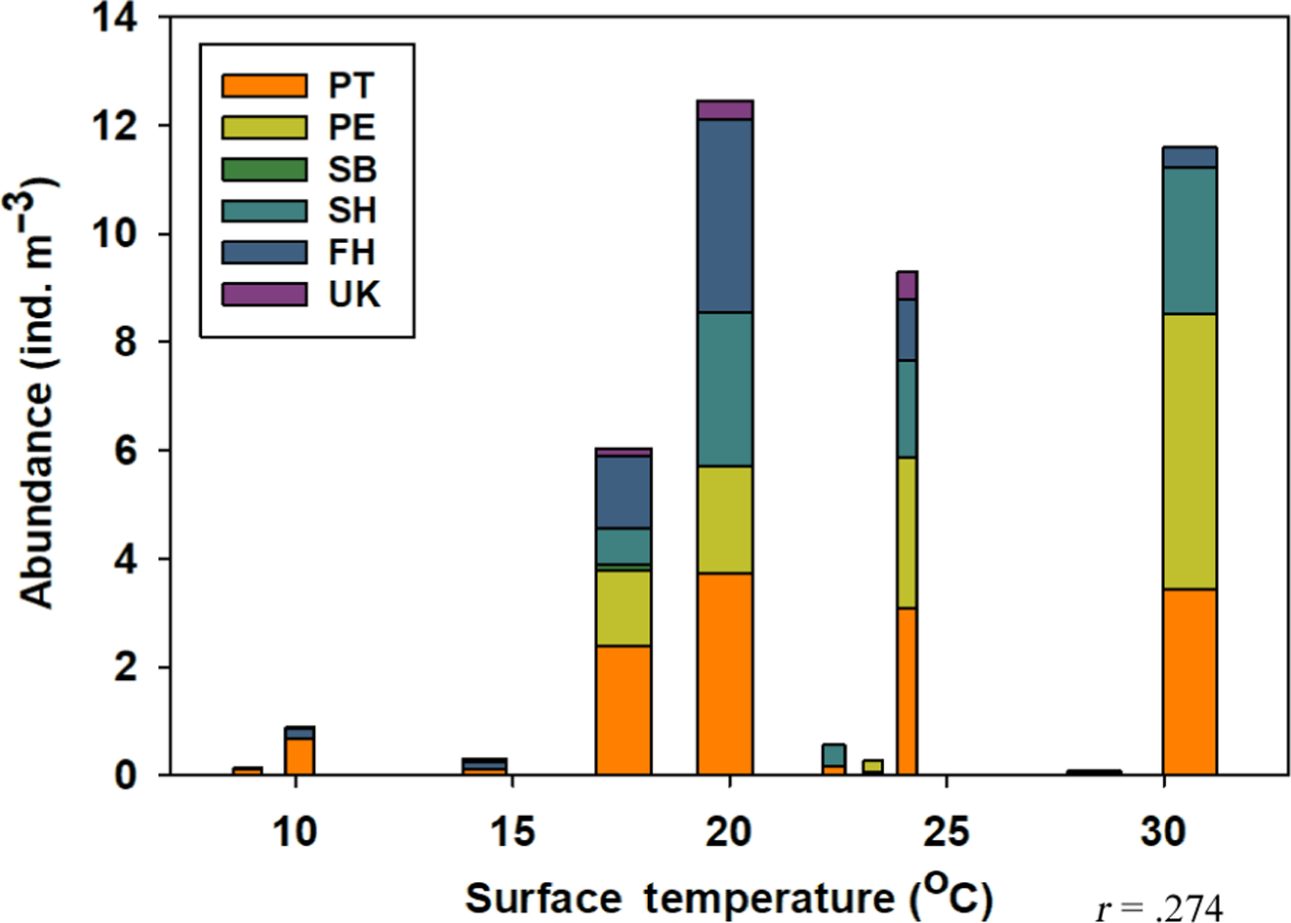
Total chaetognath abundance (*n* = 708) in individuals per m^−3^ per species and surface temperature measurements (*t*(70) = 2.3871; *p* = .0197; 95% CI [0.0456, 0.4758]). FH, *Ferosagitta hispida*; PE, *Parasagitta elegans*; PT, *Parasagitta tenuis*; SB, *Sagitta bipunctata*; SH, *Sagitta helenae*; UK, Unknown chaetognath.

Chaetognath abundance was highest between temperatures of 17°C to 25°C; this was reflected in both surface and bottom analyses (Figure 5). The Pearson correlation coefficient for surface temperature and abundance was 0.274 (*t*(70) = 2.3871; *p* = .0197; 95% CI [0.0456, 0.4758]), and bottom temperature and abundance had a correlation coefficient of 0.270 (*t*(70) = 2.3467; *p* = .0218; 95% CI [0.0410, 0.4722]). The percentage of individuals with eggs present was highest at temperatures around 24°C (Figure 4). *P. tenuis* was the only species to exhibit egg presence at surface temperatures below 13°C (Figure 4). *P. elegans* and *S. helenae* had the highest egg presence at surface temperatures around 24°C (Figure 4). The relationship between surface temperature and egg presence was positive and significant (*r* = .455; *t*(70) = 4.2748; *p* < .001; 95% CI [0.2496, 0.6212]), as was the relationship between bottom temperature and egg presence (*r* = .460; *t*(70) = 4.3346; *p* < .001; 95% CI [0.2556, 0.6251]).

**FIGURE 6:**
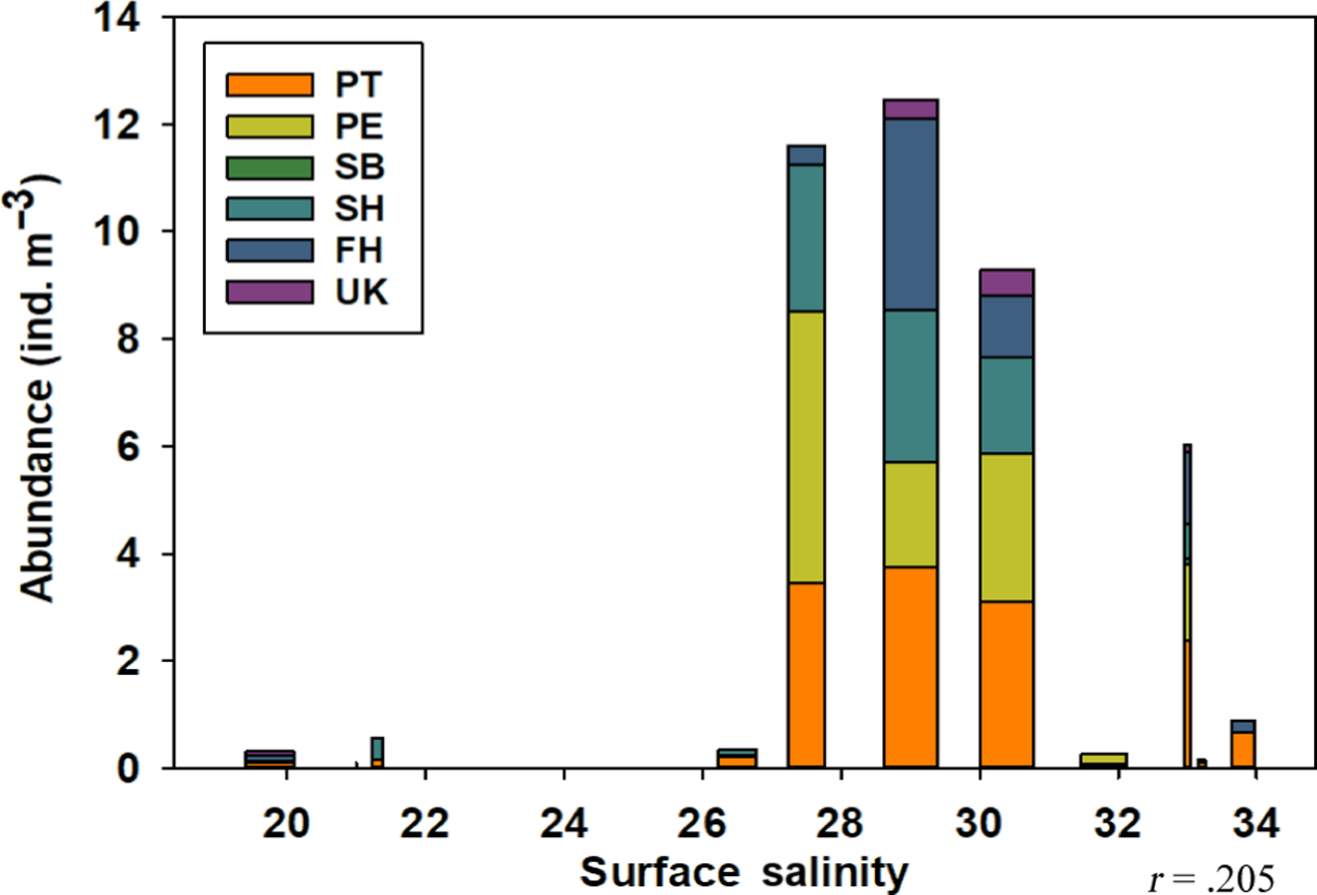
Total chaetognath abundance (*n* = 708) in individuals per m^−3^ per species and surface salinity measurements (*t*(70) = 1.7489; *p* = .0847; 95% CI [−0.0284, 0.4165]). FH, *Ferosagitta hispida*; PE, *Parasagitta elegans*; PT, *Parasagitta tenuis*; SB, *Sagitta bipunctata*; SH, *Sagitta helenae*, UK, Unknown chaetognath.

The highest abundances for all species occurred between a bottom salinity of 30 and 34, and surface salinity between 27.5 and 34 (Figure 6). The Pearson correlation coefficient for surface salinity and abundance was 0.205 (*t*(70) = 1.7489; *p* = .0847; 95% CI [−0.0284, 0.4165]), and the correlation coefficient for bottom salinity and abundance was 0.117 (*t*(70) = 0.9851; *p* = .328; 95% CI [−0.1179, 0.3394]). For surface salinity measurements, most species showed higher percentages of egg presence when salinity was greater than 25. Only one species, *F. hispida*, did not have any occurrences of eggs present in salinity below 25 for both surface and bottom measurements. The total percentage of chaetognaths with eggs present was higher for surface salinity measurements of 30 or higher. The Pearson correlation coefficient for surface salinity and egg presence was 0.274 (*t*(70) = 2.3868; *p* = .0197; 95% CI [0.0456, 04758]), and the correlation coefficient for bottom salinity and egg presence was 0.286 (*t*(70) = 2.5025; *p* = .0147; 95% CI [0.0588, 0.4860]).

**FIGURE 7:**
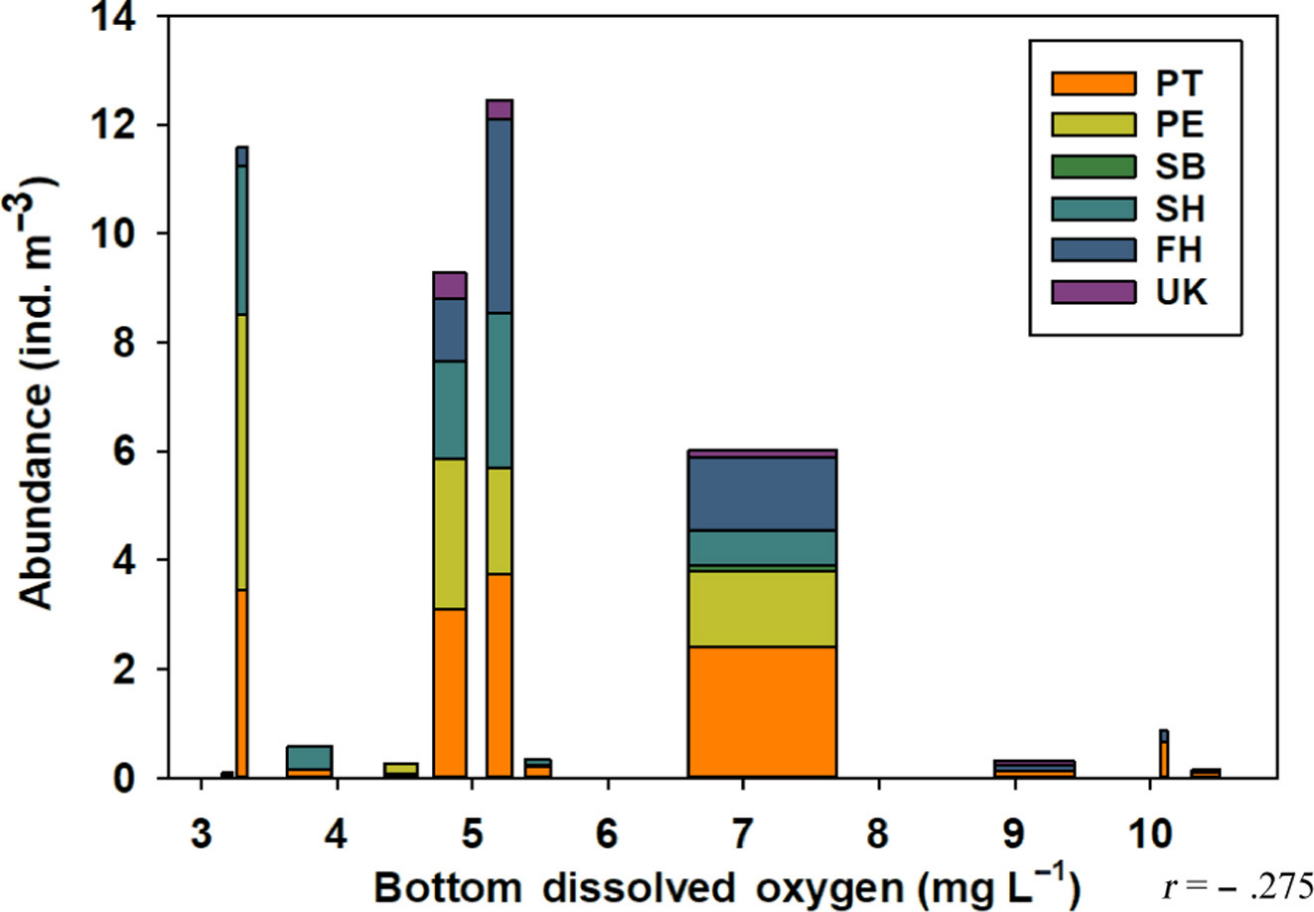
Total chaetognath abundance (*n* = 708) in individuals per m^−3^ per species and bottom dissolved oxygen measurements (*t*(70) = −2.3881; *p* = .1964; 95% CI [−0.4759, −0.0457]). FH, *Ferosagitta hispida*; PE, *Parasagitta elegans*; PT, *Parasagitta tenuis*; SB, *Sagitta bipunctata*; SH, *Sagitta helenae*; UK, Unknown chaetognath.

Higher abundances and more species were found at lower dissolved oxygen levels (Figure 7). The relationship between surface dissolved oxygen (*t*(70) = −2.268; *p* = .0264; 95% CI [−0.4651, −0.0319]), bottom dissolved oxygen (*t*(70) = −2.3881; *p* = .1964; 95% CI [−0.4759, −0.0457]), and total abundance was negative (surface *r* = −.262; bottom *r* = −.275). Surface dissolved oxygen and egg presence were moderately correlated and the relationship was significant (*r* = −.472; *p* < .001; *t*(70) = −4.4814; 95% CI [−0.6344, −0.2700]), as was the correlation between bottom dissolved oxygen and egg presence (*r* = −.536; *p* < .001; *t*(70) = −5.317; 95% CI [−0.6831, −0.3479]). Per species trends aligned with the inverse relationship for both surface and bottom dissolved oxygen measurements (Figure 4).

**FIGURE 8:**
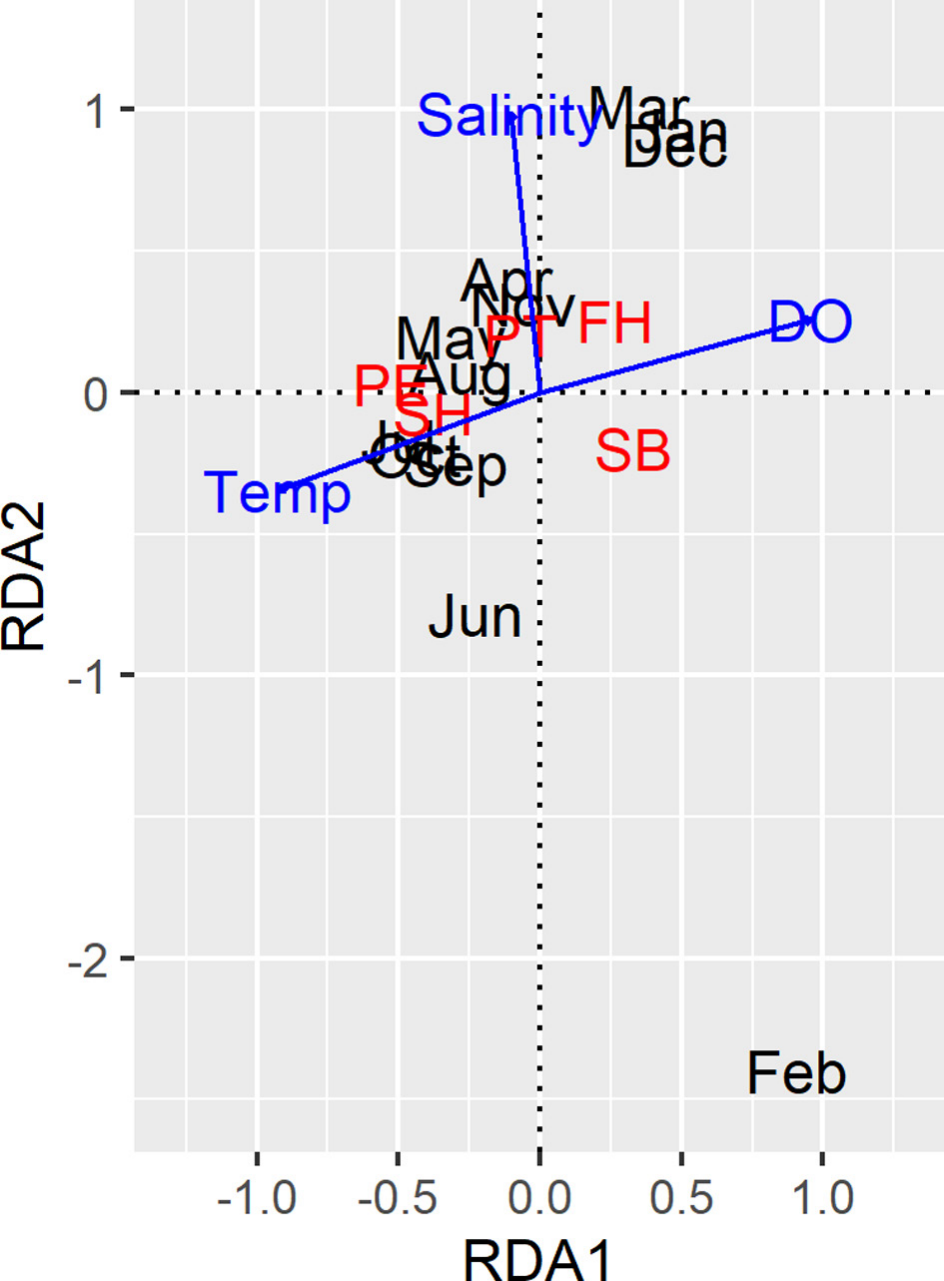
Redundancy analysis for community ordination of chaetognath abundance per species and month with environmental vectors. FH, *Ferosagitta hispida*; PE, *Parasagitta elegans*, PT, *Parasagitta tenuis*; SB, *Sagitta bipunctata*; SH, Sagitta helenae.

The RDA analysis was significant (*p* = .04) and explained 18.5% of the variation in community composition (adjusted *R* ^2^ = .185). The data were significant with the RDA1 axis (*p* = .012) and indicated surface temperature was a significant factor in determining chaetognath community composition (*p* = .002). Spring and summer months (April, May, July, August), as well as winter months (December, January) are clustered together (Figure 8), indicating similar species compositions during those periods. February is an outlier, differing along the salinity axis, with only one species present (*S. bipunctata)*.

### 3.3 Feeding rates

Using a digestion rate of 269.2 ± 74.5 min and an average number of prey per chaetognath of 2.151, the daily feeding rate per chaetognath would be 0.1918 ± 0.0102. Thus, not accounting for species composition, the average number of chaetognaths found in this study could consume 0.68 ± 0.0361 copepods per m^−3^ per day, and at the highest abundances found in this study, a maximum of 2.39 ± 0.1271 copepods per m^−3^ per day.

## 4 DISCUSSION

Chaetognath abundance and egg presence were compared with surface and bottom measurements of temperature, salinity, and dissolved oxygen levels to determine the driving abiotic factors behind chaetognath's seasonal variability and their reproductive cycles within North Inlet Estuary, South Carolina, USA. Total chaetognath abundance ranged from 0.27 ind. m^−3^ to 12.46 ind. m^−3^, which is an abundance several magnitudes lower than that found in similar regions (Coston‐Clements et al., 2009; Lonsdale & Coull, 1977). We found temperature (surface *r* = .274; bottom *r* = .270), salinity (surface *r* = .205; bottom *r* = .117), and dissolved oxygen (surface *r* = −.262; bottom *r* = −.275) all had small, non‐significant correlations to chaetognath abundance. We observed chaetognath's egg presence to be most strongly and significantly correlated with temperature (surface *r* = .455; bottom *r* = .460) and dissolved oxygen (surface *r* = −.472; bottom *r* = −.536). The relationship between salinity and egg presence had a low, non‐significant correlation (surface *r* = .274; bottom *r* = .286).

While we found a lower abundance relative to comparative studies, we observed similar intra‐annual fluctuation patterns in abundance. Previous studies conducted in North Inlet Estuary found the highest abundances of chaetognath occurred in May (134 ind. m^−3^ in 1974), July (89 ind. m^−3^ in 1975), and August (164 ind. m^−3^ in 1974; 85 ind. m^−3^ in 1975) (Lonsdale & Coull, 1977) and June, July and August (Allen et al., 2008). Owre (1960) found *S. bipunctata* had the highest abundances during summer and autumn, and *S. helenae* in the spring (April), while *F. hispida* had an October maxima in 1950 and 1951, as well as a spring maxima in 1951 in the waters of the eastern coast of Miami, Florida. The findings of this study, which noted the highest abundances during summer months, specifically April (12.46 ind. m^−3^), May (9.78 ind. m^−3^), and July (11.60 ind. m^−3,^) demonstrate similar fluctuations in abundance to such previous studies, albeit at a much lower volume. Coston‐Clements et al. (2009) study on chaetognath abundance off of the North Carolina coast identified 16 species of chaetognaths present, including all five observed in this study. Pierce & Wass (1962) found *P. tenuis* to be the second most abundant species, and identified 12 species present in the coastal waters of the southeastern USA, of which *S. bipunctata*, *S. helenae*, *P. tenuis*, and *F. hispida* were also found in this study. Coston‐Clements et al. (2009) further determined the seasonal abundance of each species varied, with the highest mean abundance of chaetognaths occurring in November (38.62 ± 13.34 ind. m^−3^). This study had moderate abundances occurring in November, specifically of *P. tenuis* (2.39 ind. m^−3^), *F. hispida* (1.34 ind. m^−3^), and *P. elegans* (1.40 ind. m^−3^).

We identified a low positive correlation between chaetognath abundance and temperature (surface *r* = .274; bottom *r* = .270). The RDA analysis indicated some seasonality in species composition and abundance, both of which varied mostly with surface temperature axis (Figure 8). Houser and Allen (1996) observed densities of chaetognaths in North Inlet Estuary were not related to salinity or depth but found a positive relationship between chaetognath abundance and water temperature. Similarly, we observed the percentage of individuals with eggs present was moderately correlated with temperature (surface *r* = .455; bottom *r* = .460). This finding aligns with previous studies, which identified temperature as a significant driver in juvenile abundance (Nogueira Júnior et al., 2019), as well as egg development and laying times (reviewed in Alvariño, 1990), with laying occurring earlier in the day on warm days and most species breeding primarily in spring, although noting breeding does occur year‐round in tropical waters. Spawning times appear to vary by species, with *P. elegans* and *P. tenuis* seemingly spawning year‐round. Zo (1973) found *P. elegans* had two seasonal egg development periods, spring and autumn, where high abundances of eggs were present. The highest abundances of eggs present in Bedford Basin, Nova Scotia, occurred from March to June, as well as September to December (Zo, 1973). Similarly, this study found *P. elegans* highest period of egg presence occurred in spring and summer (April, May, and July) as well as fall (October and November). Owre (1960) found *S. bipunctata* did not have year‐round spawning times, mainly reproducing in the early spring, summer, or in late winter, and *S. helenae* and *F. hispida* did reproduce year‐round, with *S. helenae* having an accelerated breeding rate during May and June, while *F. hispida* had the highest reproduction rates in mid‐winter and spring. These findings support the data collected in this study, which found *F. hispida* to have the highest egg presence rates occurring in spring (April) and *S. helenae*'s egg presence peaking in summer (July). There were not enough individuals found in this study to make conclusions regarding *S. bipunctata*'s reproductive seasonality.

Salinity had a slight positive correlation with both chaetognath abundance (surface *r* = .205; bottom *r* = .117) and egg presence (surface *r* = .274; bottom *r* = .286). As chaetognaths have weak osmoregulatory functioning, salinity is an important factor in their geographic distribution and their tolerance to low salinity levels directly influences their presence in estuaries (Bieri, 1959; Coston‐Clements et al., 2009; Nogueira Júnior et al., 2019; Owre, 1960; Pierce, 1951; Pierce & Wass, 1962; Ulloa et al., 2000). While this study found weak correlations between salinity and chaetognath abundance, primarily oceanic species (such as *F. enflata*, *S. bipunctata*, *and Sagitta megalopthalma*) have demonstrated significant positive relationships between chaetognath assemblages and salinity levels (Gilmartin et al., 2020). This likely indicates the species found in this study have higher tolerances to wide salinity fluctuations, which would account for the very low abundance of *S. bipunctata* observed in North Inlet Estuary. Pierce and Wass (1962) found *P. tenuis* and *S. helenae* demonstrated a wide temperature and salinity tolerance, whereas *F. hispida* demonstrated salinity tolerance up to 36. Similarly, Pierce and Wass (1962) found *S. bipunctata* was more common in salinities under 36, though could be found in wide salinity fluctuations, and suggested that its principal recruitment in Carolinian coastal waters was due to the Florida Current. The extreme monthly fluctuations in total chaetognath abundance are likely due to shifts in water mass, and not actual fluctuations in population. For instance, the decline in total chaetognath abundance which occurred in June coincides with a decrease in salinity (from 30.4 to 21.3 at surface measurements and from 30.5 to 24.7 at bottom measurements), indicating increased freshwater runoff from the estuary's watershed and reduced percentage of oceanic water in the sample. Similarly, August saw lower salinity levels (21 at surface measurements and 25 at bottom measurements) which also coincided with a decrease in chaetognaths found in the sampling area. This indicates major runoff or precipitation events likely drive salinity below chaetognath tolerance thresholds, driving them out of the estuary and into more coastal waters.

Higher chaetognath abundances and more species were found at lower dissolved oxygen levels. It is possible chaetognaths prefer lower oxygen concentrations, as low dissolved oxygen may not impose any physiological costs while correlating with another factor chaetognath's need for survival. However, as dissolved oxygen was the environmental factor most strongly (surface *r* = −.472; bottom *r* = −.536) and significantly (*p* < .001) negatively correlated with chaetognath's egg presence, it is somewhat unlikely chaetognaths prefer lower dissolved oxygen concentrations. Rather, as temperature had similar correlation coefficient values as dissolved oxygen, it could be changes in temperature driving changes in chaetognath abundance and egg presence, and the significance of dissolved oxygen is a side effect of the two environmental factor's correlation to each other. However, both Besiktepe & Unsal (2000) and Saltzman & Wishner (1997) found adult chaetognaths are capable of tolerating a wide range of oxygen concentrations, and can occur below the oxygen minimum zone, although are typically found in higher abundances at shallower depths. It is important to note that in this study, it did not appear low dissolved oxygen concentrations (<4 mg/L) negatively impacted chaetognath abundance, although the threshold at which they are negatively affected may not have been observed.

Chaetognaths play an important role in marine trophic interactions and understanding their seasonality is important for our understanding of copepod populations. The impact of chaetognaths on copepod populations was estimated by Baier and Purcell (1997), who found that chaetognaths can consume up to 44% of large copepod standing stocks per day over the mid‐shelf area of the southeastern USA. Average abundances of copepods in the studied region of North Inlet Estuary are ~7000 copepods m^−3^ (Stone and Allen, unpublished data), meaning chaetognaths in this study on average consume 0.010% of copepod standing stock each day, and at their most abundant, could consume 0.034%. As this number does not account for variations in chaetognath species composition, it is important to note that our estimations include an element of error, as consumption and digestion rates can vary between species (Feigenbaum & Maris, 1984). While chaetognaths in some systems can exert large top‐down pressure on the copepod community (Tönnesson and Tiselius, 2005), based on our calculated feeding rates and abundances, we found no evidence for high predation pressure, on average, in this system. However, as total abundances of chaetognaths in North Inlet Estuary has been reported much higher than those observed in this study (>150 ind. m^−3^, Allen et al. 2008), chaetognaths may exert top‐down control on copepods during these periods of higher abundances.

There are several hydrographic and behavioral factors that might have influenced the results of this study. Changes in predation, parasites, and viruses may have impacted the chaetognath population in North Inlet Estuary. Additionally, changes in environmental variables such as water runoff and temperature may have impacted our results. While the study site has experienced long‐term warming, specifically in winter months, salinity in the estuary has been variable but has not changed significantly in the long term, indicating relatively stable levels of freshwater run‐off in the system (Allen et al., 2014). However, short term fluctuations in salinity due to increased run‐off likely did affect chaetognath assemblages in the system. Further, diel vertical migration, in which organisms migrate vertically through the water column for prey, protection, or resource partitioning, has been shown to impact chaetognath abundance (Sweatt and Forward, 1985). However, diel vertical migration likely played little role in the observed chaetognath abundances in this study, as chaetognath abundance in North Inlet Estuary is greatest during the daytime and not related to depth (Houser and Allen, 1996).

A potential cause for the relatively low levels of chaetognath abundance found in this study could lie within the sampling design. While this study included one sampling location within an estuary at a shallow depth (~2 m), similar studies in the region sampled at a variety of different depth locations, with some stations located roughly 200 miles from shore (Coston‐Clements et al., 2009; Pierce & Wass, 1962). These differences in sampling designs are likely major sources of uncertainty in this study and may convolute comparisons between this study and research in similar regions.

The low abundance seen in this study, however, may still be an accurate representation of chaetognath presence in the estuary. Time‐series collections utilizing similar methodologies indicate mean chaetognath abundances in North Inlet Estuary have been historically lower than other regional studies, with a long‐term average abundance of 8 ind. m^−3^ over the period of 1981–2003 (Allen et al., 2008). Additionally, during this period there was an observed 26% decrease in chaetognath abundance compared to the long‐term mean (Allen et al., 2008). This could indicate that the low numbers found in this study is representative of the estuary, and while the estuary experiences occasional large spikes in abundance (>150 ind. m^−3^ observed in Allen et al., 2008), chaetognath abundances are typically low.

The availability of copepods and other prey organisms may have influenced the presence of chaetognaths at our study site. As estuaries are highly variable in their chemical conditions, environmental conditions and changes in water mass may have further impacted the sampling design. Sampling at mid‐tide, when salinity levels are lower, may have impacted the amount and species composition of chaetognaths found. It is likely the species found in the North Inlet Estuary can tolerate wide fluctuations in salinity levels, as chaetognaths were found in a wide range of salinities (19.8–34.2). There may be other species of chaetognath present in the North Inlet‐Winyah Bay area that are dominant during high tide or located closer to the mouth of the estuary, conditions unaccounted for in this study's sampling design. Further sampling at high tide would provide an interesting comparison to the current sampling design and sampling closer to the mouth of the estuary might provide different results in species composition and concentrations of egg presence within chaetognaths. While outside the scope of this study, genetic analyses, specifically metabarcoding, would be beneficial in identifying the unknown chaetognaths found and providing a better understanding of species composition in the estuary. Future analyses should be done on a multi‐year basis to better understand chaetognath seasonality and the relationship between their abundance and environmental factors.

While limited in scope, our findings provide an initial foray into analyzing chaetognath abundance and reproductive seasonality in North Inlet Estuary, USA. The results of this study align with previously observed seasonal fluctuations in abundance in the estuary (Allen et al., 2008; Lonsdale & Coull, 1977) and provide the first known record of chaetognath species composition in the North Inlet‐Winyah Bay area. Our findings highlight the potential influence of environmental factors on chaetognath abundance and reproductive timing, addressing a knowledge gap in the ecology of this important zooplankton phyla's seasonality. Additional research is needed considering the impact of climate change‐induced environmental variability on chaetognath assemblages in the North Inlet‐Winyah Bay area.

## AUTHOR CONTRIBUTIONS

**Sarah E. Stone:** Conceptualization (supporting); data curation (lead); formal analysis (lead); investigation (lead); project administration (lead); software (lead); visualization (supporting); writing – original draft (lead); writing – review and editing (lead). **Joshua P. Stone:** Conceptualization (lead); formal analysis (supporting); methodology (equal); resources (supporting); visualization (lead); writing – review and editing (supporting).

## CONFLICT OF INTEREST STATEMENT

The authors declare that they have no known competing financial interests or personal relationships that could have appeared to influence the work reported in this paper.

## Data Availability

The data that support the findings of this study are openly available in Dryad at doi: 10.5061/dryad.4qrfj6qft.
